# Relation Between Thickness and TFTs Properties of HfO_2_ Dielectric Layer Synthesized by Plasma-Enhanced Atomic Layer Deposition

**DOI:** 10.3390/nano15100719

**Published:** 2025-05-10

**Authors:** Qizhen Chen, Wanqiang Fu, Jing Han, Xiaoying Zhang, Shui-Yang Lien

**Affiliations:** 1 Xiamen Key Laboratory of Development and Application for Advanced Semiconductor Coating Technology, School of Opto-Electronic and Communication Engineering, Xiamen University of Technology, Xiamen 361024, China; chenqizhen@xmut.edu.cn (Q.C.); 2222031241@stu.xmut.edu.cn (W.F.); 2022031149@stu.xmut.edu.cn (J.H.); xyzhang@xmut.edu.cn (X.Z.); 2 Department of Materials Science and Engineering, Da-Yeh University, Dacun, Changhua 51591, Taiwan

**Keywords:** high k, gate insulator, film thickness, low leak current

## Abstract

The advancement of portable high-definition organic light-emitting diode (OLED) displays necessitates thin film transistors (TFTs) with low power consumption and high pixel density. Amorphous indium gallium zinc oxide (a-IGZO) TFTs are promising candidates to meet these requirements. However, conventional silicon dioxide gate insulators provide limited channel modulation due to their low dielectric constant, while alternative high-k dielectrics often suffer from high leakage currents and poor surface quality. Plasma-enhanced atomic layer deposition (PEALD) enables the atomic-level control of film thickness, resulting in high-quality films with superior conformality and uniformity. In this work, a systematic investigation was conducted on the properties of HfO_2_ films and the electrical characteristics of a-IGZO TFTs with different HfO_2_ thicknesses. A *Vth* of −0.9 V, *μ_sat_* of 6.76 cm^2^/Vs, *SS* of 0.084 V/decade, and *I_on_*/*I_off_* of 1.35 × 10^9^ are obtained for IGZO TFTs with 40 nm HfO_2_. It is believed that the IGZO TFTs based on a HfO_2_ gate insulating layer and prepared by PEALD can improve electrical performance.

## 1. Introduction

Active-matrix organic light-emitting diodes (AMOLEDs) are a leading display technology, consisting of organic light-emitting pixels, thin film transistors (TFTs), and storage capacitance [1,2,3,4,5]. Among these components, the performance of TFTs is particularly critical for AMOLED functionality, driving extensive research into optimizing their channel layers. Amorphous indium gallium zinc oxide (a-IGZO) TFTs have become highly attractive as promising candidates for switching and driving devices in active-matrix electronics due to their high carrier mobility, large switching current ratio, excellent uniformity, and optical transparency in the visible spectrum [6,7,8]. While significant attention has been given to improving channel layer properties, the role of gate insulators is equally crucial. The characteristics of the gate insulator directly influence the threshold voltage (*Vth*), subthreshold swing (*SS*), and overall electrical stability of TFTs.

Conventional gate dielectrics such as silicon dioxide (SiO_2_) and silicon nitride (Si_3_N_4_) are limited by their relatively low dielectric constants (*k*), which lead to weak channel modulation and require high driving voltages [9,10,11]. These shortcomings result in increased power consumption and reduced device lifespan, particularly in next-generation flexible and high-resolution displays. To address these challenges, two major strategies have been proposed: (1) reducing the thickness of gate insulators and (2) employing high-*k* dielectric materials. While thinner dielectric films can lower operating voltages, they often suffer from degraded quality and increased leakage currents. Adopting high-*k* gate dielectric materials can minimize the physical thickness of a gate insulator to achieve a high current with a low operating voltage, because of the large gate capacitance. Hafnium oxide (HfO_2_) is a particularly attractive high-k dielectric due to its thermodynamic stability, high dielectric constant, and excellent electrical performance under high drive currents. However, challenges such as high leakage current, poor surface morphology, and the formation of trap states at the dielectric/channel interface still hinder its practical application in TFTs.

Therefore, optimizing both the thickness and the interfacial quality of HfO_2_ films is critical for enhancing TFT device performance. Various deposition methods have been explored for fabricating high-*k* dielectric layers. Although methods such as molecular beam epitaxy (MBE) and chemical vapor deposition (CVD) offer high deposition rates, their high processing temperatures limit their applicability to the flexible substrates and low-temperature processes required for display integration [12,13,14]. Atomic layer deposition (ALD) enables atomic-scale thickness control and excellent step coverage, making it suitable for ultrathin, conformal dielectric films. Plasma-enhanced atomic layer deposition (PEALD), a variant of ALD, further improves film quality by providing higher film density, smoother surfaces, and better interfacial characteristics due to enhanced plasma reactivity. These advantages contribute to reduced interface trap density, improved *SS*, enhanced carrier mobility, and lower leakage currents in oxide TFTs—making PEALD a highly promising technique for advanced electronics.

In this work, the effect of HfO_2_ film thickness was systematically investigated, with deposition by PEALD, including the optical and electrical properties of the dielectric layer and the performance of a-IGZO TFTs. HfO_2_ films with thicknesses of 20, 40, 60, and 80 nm were deposited, and their influence on capacitance, leakage current, and device switching behavior was comprehensively evaluated. This work provides new insights into thickness optimization strategies for high-*k* dielectrics in TFT applications and highlights the potential of PEALD-grown HfO_2_ films for next-generation electronics.

## 2. Materials and Methods

Heavily doped p-type (100) silicon wafers (p++-Si, ρ < 0.001 Ω·cm) (Shandong Zhuojing Electronic Technology Co., Ltd., Jinan, China) were used as both the substrate and gate electrode. The wafers were diced into 2.5 × 2.5 cm^2^ pieces and underwent a three-step cleaning process to ensure surface preparation. First, the substrates were soaked in deionized water for 10 s, followed by immersion in a 2% diluted hydrofluoric acid solution (Sinopharm Chemical Reagent Co., Ltd., Shanghai, China) for 1 min, and finally rinsed again in deionized water for 10 s. After cleaning, the wafers were immediately dried with a nitrogen blower and transferred into the reaction chamber of PEALD. HfO_2_ thin films with thicknesses of 20, 40, 60, and 80 nm were deposited using tetrakis(ethylmethylamino)hafnium (TEMAH, purity: 99.9999%, AimouYuan, Nanjing, China) and O_2_/Ar plasma (produced in a microwave cavity by an inductive coupling of radio frequency (RF) power (Litmas RPS, Advanced Energy, Denver, CO, USA)) in a PEALD system (Picosun R-200, Espoo, Finland) at temperatures ranging from 100 to 450 °C, with a plasma power of 2500 W; each ALD cycle consisted of a 1.6 s TEMAH pulse, 10 s N_2_ purge, 10 s plasma exposure, and 12 s N_2_ purge [15]. A 30 nm amorphous indium gallium zinc oxide (a-IGZO) channel layer, with a 35% In_2_O_3_ cycle ratio, was also deposited using the same PEALD system following previously reported procedures [16]. The IGZO thin films were fabricated by stacking PEALD-grown ZnO, Ga_2_O_3_, and In_2_O_3_ sub-layers using DEZ, TMGa, and InCp (Nanjing Aimuyuan Scientific Equipment Co., Ltd., Nanjing, China) as respective precursors, with an O_2_ plasma power of 2500 W and a deposition temperature of 250 °C. The active layer was patterned into a square shape with dimensions of 1000 μm. The aluminum source and drain electrode with a thickness of 150 nm were prepared by thermal vacuum deposition, and a shadow mask (Shenzhen Kebaoyuan Technology Co., Ltd., Shenzhen, China) was employed to create a patterned channel with dimensions of 80 μm in length and 1000 μm in width.

The capacitance characteristics of the HfO_2_ film and electronic characteristics of the a-IGZO TFT based on HfO_2_ film were measured by a semiconductor parameter analyzer (200-SCS, Keithley Instruments Inc., Beaverton, OR, USA) at room temperature under an ambient atmosphere. The thickness of the HfO_2_ film was determined using a step profiler (D-500, KLA-Tencor Corp., Milpitas, CA, USA). The crystalline structure of HfO_2_ films was analyzed via grazing incident X-ray diffraction (GIXRD, TTRAX III, Rigaku Corp., Ibaraki, Japan). The root-mean-square (RMS) surface roughness was assessed using an atomic force microscope (AFM, XE7, Park Systems Corp., Suwon, Republic of Korea). The surface morphologies of HfO_2_ films were examined using field emission scanning electron microscopy (FESEM, Sigma 500, Carl Zeiss AG, Oberkochen, Germany). Additionally, the cross-section images of the a-IGZO TFT device were scrutinized using field emission transmission electron microscopy (FE-TEM, JEM-2100F, JEOL Ltd., Tokyo, Japan).

## 3. Results and Discussion

**Figure 1:**
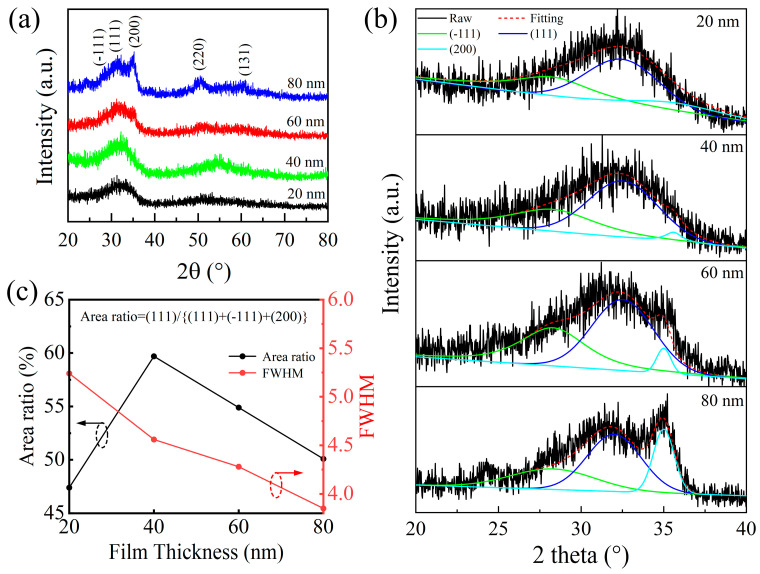
(**a**) GIXRD patterns of the HfO_2_ films with varying thickness; (**b**) the deconvolution of GIXRD peaks for HfO_2_ films g with different thicknesses; (**c**) area ratio and FWHM of the (111) peaks.

The thickness of the gate insulator and the quality of its interface are critical factors influencing TFT performance. To assess the structural properties of the HfO_2_ films, GIXRD measurements were performed on samples with varying thicknesses, as shown in Figure 1a. Diffraction peaks were observed at 2θ = 28.31°, 31.44°, and 35.60°, corresponding to the (−111), (111), and (200) planes, respectively, as indexed for HfO_2_ in the standard JCPDS file (data No. 06-0318). As the film thickness increases from 20 to 80 nm, the overall crystallinity improves, evidenced by the increasing intensity of the diffraction peaks. The detailed GIXRD spectra of the HfO_2_ films with varying thickness were deconvoluted, as shown in Figure 1b. The intensities of all diffraction peaks increase with film thickness, with particular emphasis on the (111) peak, as it significantly influences the dielectric constant of the films [17,18,19]. The (111) peak area ratio relative to the total diffraction intensity, plotted in Figure 1c, reaches a maximum at 40 nm and then declines, suggesting an optimal crystalline orientation at this thickness. Moreover, the full width at half-maximum (FWHM) of the (111) peak decreases with increasing thickness, indicating reduced lattice disorder and improved grain quality. Specifically, when the film thickness is small, surface energy dominates, and the film tends to form an amorphous or low-crystallinity structure [20,21,22,23,24]. As the film thickens, grain growth and packing density are enhanced, resulting in improved crystallinity. Notably, the 40 nm HfO_2_ film demonstrates the most favorable structural characteristics, which are expected to translate into superior dielectric performance.

**Figure 2:**
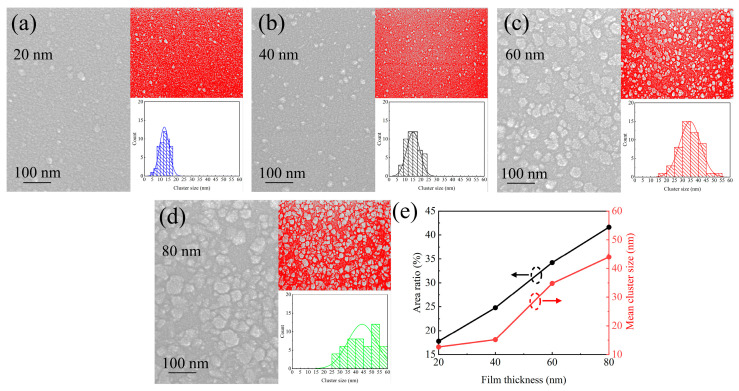
SEM images of HfO_2_ films with varying thicknesses: (**a**) 20 nm; (**b**) 40 nm; (**c**) 60 nm and (**d**) 80 nm. The upper inset highlights the white-colored regions used for grain boundary area computation via ImageJ software, while the lower inset presents the cluster size distribution, (**e**) The grain/cluster area ratio and mean cluster size corresponding to the thickness.

FESEM was employed to analyze the surface morphology of HfO_2_ films with varying thicknesses, as shown in Figure 2. The 20 nm film (Figure 2a) displays small, uniformly distributed grains/clusters, with size statistics shown in the inset. As the film thickness increases (Figure 2b–d), both the grain/cluster size and coverage area expand. Quantitative analysis using ImageJ software (version 1.54f, developed by the National Institutes of Health, Bethesda, MD, USA) shows that the grain/cluster area ratio increases from 17.78% at 20 nm to 41.61% at 80 nm, while the mean cluster size grows from 12.64 nm to 43.99 nm (Figure 2e). Interestingly, the 40 nm film shows only a modest increase in grain size compared to the 20 nm film, suggesting a transitional regime in morphology evolution.

**Figure 3:**
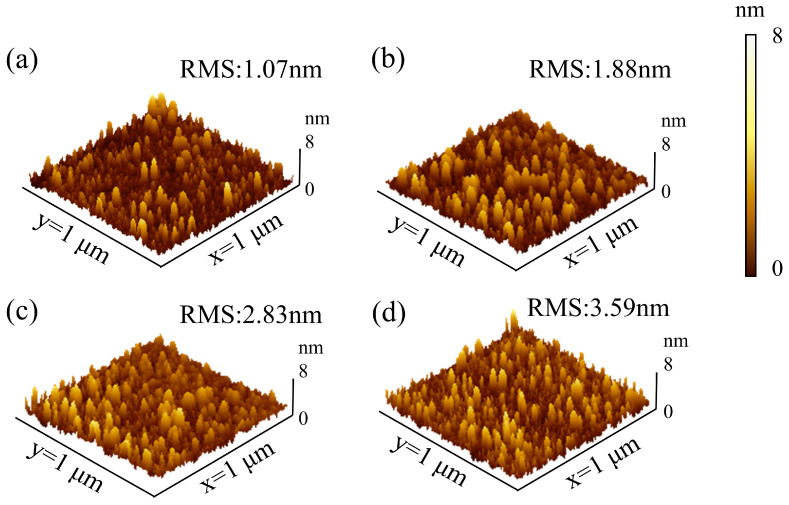
AFM images of the HfO_2_ films with varying thicknesses: (**a**) 20; (**b**) 40; (**c**) 60 and (**d**) 80 nm.

Complementary AFM measurements (Figure 3a–d) further confirm the surface morphology trends. The root-mean-square (RMS) roughness increases progressively with thickness, from 1.07 nm at 20 nm to 3.59 nm at 80 nm, consistent with the growth of grains/clusters observed in SEM. Notably, the 20 nm and 40 nm films exhibit relatively smooth and continuous surfaces with low roughness and minimal island formation, while the 60 nm and 80 nm films develop more pronounced surface textures and grain boundaries. These findings indicate that the surface morphology of HfO_2_ evolves significantly with thickness, with smoother, denser films at thinner regions and rougher, more granular structures at greater thicknesses.

**Figure 4:**
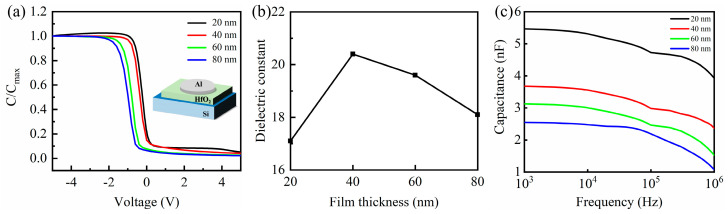
HfO_2_ MOS capacitors at different thicknesses: (**a**) C–V curve; (**b**) the relationship between the thickness of HfO_2_ films and the dielectric constant k; (**c**) Capacitance versus frequency for HfO_2_ thin films measured from 1 kHz to 1 MHz.

The capacitance and dielectric constant (*k*) are key parameters that directly affect the electrical performance of the gate insulator in TFTs, and both are influenced by film thickness and interfacial quality. Figure 4a presents the C–V curves of n-Si/HfO_2_/Al MOS capacitors measured at 1 MHz for HfO_2_ films with varying thicknesses. The capacitance in the accumulation region was extracted from the voltage range of −5 V to +10 V, corresponding to the maximum capacitance (*C_max_*). The measured *C_max_* values were 3.88 × 10^−9^ F, 2.36 × 10^−9^ F, 1.53 × 10^−9^ F, and 1.06 × 10^−9^ F for HfO_2_ thicknesses of 20, 40, 60, and 80 nm, respectively. Figure 4b shows the relationship between film thickness and the dielectric constant *k*, which is calculated using the following equation [25,26]:

(1) $$k=\frac{C_{max}}{A}\frac{d}{\varepsilon_{0}}$$

where *d* stands for the thickness of the HfO_2_ film, *A* is the area of the capacitor (5.28 × 10^−7^ m^2^), and *ε*_0_ is the dielectric constant of the vacuum. Capacitance per unit area (*C_ox_*) was calculated as 73.5, 44.7, 29.0, and 20.1 nF/cm^2^ for 20, 40, 60, and 80 nm, respectively, exhibiting a decreasing trend with increasing thickness. The calculated *k* values initially increased from 17.1 (20 nm) to a peak of 20.4 (40 nm) and then declined to 18.1 (80 nm). This trend is attributed to the improved crystallinity and packing density at intermediate thicknesses, particularly the enhanced (111) orientation at 40 nm, which is known for its high polarizability [17,18,19]. Beyond 40 nm, although crystallinity continues to improve, the relative intensity of the (111) plane decreases (as shown in Figure 1c), leading to a reduction in the effective dielectric constant. To further evaluate the frequency-dependent behavior of the HfO_2_ films, capacitance–frequency (C–*f*) measurements were conducted for samples with thicknesses of 20, 40, 60, and 80 nm, as shown in Figure 4c. The capacitance (C) was extracted from the accumulation region in the frequency range of 1 kHz to 1 MHz. All samples exhibited relatively stable capacitance values across the measured frequencies, indicating good dielectric reliability and low interface trap density. The 20 nm film showed the highest capacitance, while the 40 nm film exhibited the smallest variation with frequency, suggesting the best overall dielectric and interface stability at this thickness. The fixed charge (*Q_f_*) is determined from the C–V characteristics, as reported in [27,28]:

(2) $$Q_{f}=\frac{{(\varphi }_{ms}-V_{FB})C_{ox}}{q\;A}$$

*φ_ms_* (0.3 eV) represents the work function difference between the metal and the semiconductor, while *V_FB_* denotes the flat band voltage. Although *C_ox_* decreases with thickness, *Q_f_* increases due to a more negative shift in *V_FB_* (from −0.20 V at 20 nm to −0.97 V at 80 nm), indicating the accumulation of negative fixed charges and bulk defects in thicker films. The interface charge density (*D_it_*) was calculated from the C–V characteristics using the following expression [29,30]:

(3) $$D_{it}=\frac{2\omega {C_{ox}}^{2}G_{max}}{qA({G_{max}}^{2}+\omega^{2}{\left(C_{ox}-C_{m}G_{max}\right)}^{2})}$$

The maximum conductance, *G_max_*, and the measured capacitance, *C_m_*, at an angular frequency *ω* are considered in this expression. The 20 nm HfO_2_ film exhibits an amorphous structure, which is advantageous for the passivation of Si surfaces, thereby minimizing the *D_it_*. Table 1 summarizes the extracted values of *D_it_* and *Q_f_* for all film thicknesses, both of which increase with thickness and are strongly correlated with the degradation in TFT electrical parameters such as *Vth*, *μ_sat_*, and *SS*.

**Figure 5:**
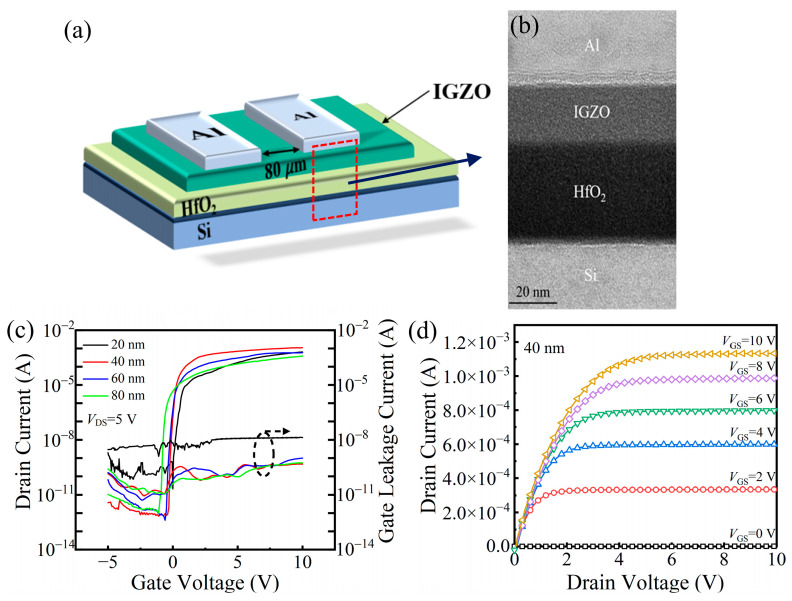
(**a**) Three-dimensional schematic diagram of IGZO-TFTs; (**b**) cross-section TEM images of IGZO TFT device with 40 nm HfO_2_ film; (**c**) transfer curves and the leakage current of a-IGZO TFT devices with different HfO_2_ thicknesses; (**d**) output characteristics of the a-IGZO TFT with a 40 nm HfO_2_ gate dielectric measured under different *V_GS_* values (*V_GS_* = 0–10 V, step = 2 V).

To evaluate the electronic performance of TFTs with different HfO_2_ thicknesses, devices with the Si/HfO_2_/IGZO/Al structure were prepared, and their 3D schematic diagram is presented in Figure 5a. To verify the interface quality between the HfO_2_ gate insulator and the IGZO channel layer, cross-sectional FE-TEM was performed on the TFT with a 40 nm HfO_2_ layer, as shown in Figure 5b. The observed uniform interface is favorable for minimizing the subthreshold swing (*SS*) by suppressing interfacial trap states. The transfer characteristics, shown in Figure 5c, exhibit typical n-type behavior. Measurements were performed at a drain-source voltage (*V_DS_*) of 5 V, while the gate voltage (*V_GS_*) was swept from −5 V to 10 V. The on/off current ratio (*I_on_*/*I_off_*) is negatively related to the thickness, and it decreased from 1.35 × 10^9^ to 3.32 × 10^8^ as the thickness increased from 20 nm to 80 nm. The *Vth* shifts negatively with increasing thickness, measuring −0.1 V, −0.2 V, −0.4, and −1.1 V for thicknesses of 20 to 80 nm, respectively, corresponding with the *Q_f_*. The *SS* was extracted using the standard method [31]:

(4) $$SS=\frac{{dV}_{GS}}{{d(log}_{10}I_{DS})}$$

The saturation field-effect mobility (*μ_sat_*) was calculated using the following equation:

(5) $$I_{DS}=\frac{W}{2L}\mu_{sat}C_{ox}{(V}_{GS}-V_{th}{)}^{2}$$

where *W* and *L* are the channel width and length. This method follows the standard extraction approach reported in [32]. The device with 40 nm HfO_2_ film demonstrates an exceptionally low *SS* value of 0.084 V/decade; meanwhile, the *μ_sat_* of the device is 6.76 cm^2^/Vs. These improvements are attributed to a reduction in interfacial trap density (*N_t_*), which was calculated using the following equation [33,34,35]:

(6) $$N_{t}=\left(\frac{SSlog_{10^{e}}}{\frac{kBT}{q}}-1\right)\frac{C_{ox}}{q}$$

where *q* is the electron charge, *k_B_* is Boltzmann’s constant, and *T* is the absolute temperature. The minimum *N_t_* value of 1.14 × 10^12^ cm^−2^ was observed at 40 nm, indicating enhanced carrier transport at the HfO_2_/IGZO interface. Meanwhile, the leakage current of the device is also shown in Figure 5c, further demonstrating the compactness of the HfO_2_ film and overcoming the inherent defects in most high-k dielectrics. Figure 5d shows the output characteristics of the a-IGZO TFT with a 40 nm HfO_2_ gate dielectric. The device exhibits well-defined linear and saturation regions, and the smooth increase in drain current with gate bias confirms effective gate modulation and good ohmic contact. These results support the superior performance observed at the 40 nm dielectric thickness. The extracted electrical parameters for all devices are summarized in Table 1. The electrical parameters of the a-IGZO TFTs exhibit clear trends with respect to HfO_2_ thickness. The *Vth* progressively shifts in the negative direction as thickness increases. Both the *μ_sat_* and on/off current ratio improve up to 40 nm, after which they begin to decline. Similarly, the *SS* reaches a minimum at 40 nm, confirming improved interfacial quality at this thickness. These results suggest that moderate dielectric thickness offers a favorable balance between interface quality and gate modulation efficiency, while excessively thick films introduce trap states and reduce gate controllability. In contrast, the 20 nm HfO_2_ film exhibits inferior performance due to its predominantly amorphous structure, which leads to a higher leakage current and a lower dielectric constant. These observations demonstrate that a moderate dielectric thickness offers an optimal trade-off between gate capacitance, interfacial quality, and electrical stability. Compared with the previous work on a transparent IGZO TFT prepared by PEALD and PEALD-deposited aluminum hafnium mixed oxide dielectrics for a-IGZO TFTs, the high-dielectric-constant insulation layer and high-quality surface morphology enable the device to achieve a higher *I_on_*/*I_off_* and an ideal *SS* [36]. To evaluate the large-area uniformity of device performance, five 2.5 × 2.5 cm^2^ substrates were placed at representative positions within the 8-inch loading zone of the PEALD chamber: top-left, top-right, bottom-left, bottom-right, and center. On each substrate, a 5 × 5 array of a-IGZO TFTs (25 devices) was fabricated. One device was randomly selected from each array for electrical testing. As summarized in Table 1, the *μ_sat_* and *SS* exhibited standard deviations within 10%, confirming excellent reproducibility and uniformity across the deposition area. These results suggest that the polycrystalline morphology of the HfO_2_ films does not significantly impact device consistency over large areas.

**Figure 6:**
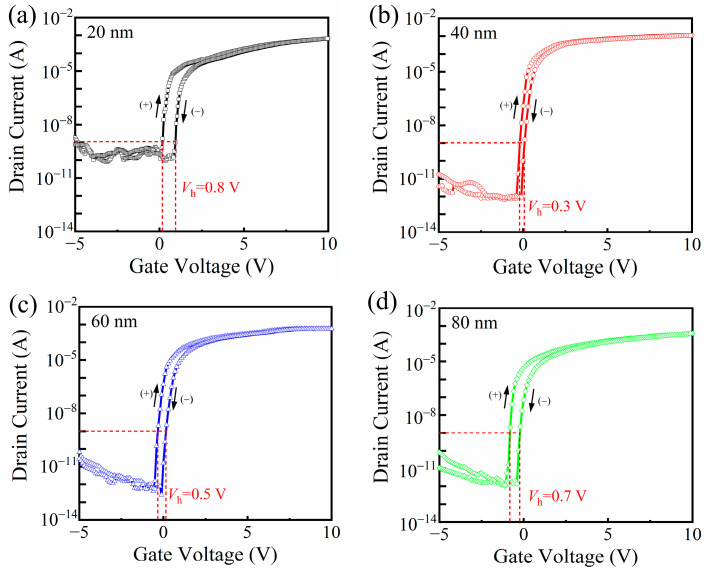
Dual-swept transfer curves of IGZO-TFTs with the HfO_2_ thickness of (**a**) 20 nm; (**b**) 40 nm; (**c**) 60 nm and (**d**) 80 nm.

To further evaluate the electrical stability of the IGZO TFTs, the transfer characteristics under both forward and reverse gate voltage sweeps were measured, as shown in Figure 6. The hysteresis voltages (*V_h_* = *V_reverse_* − *V_forward_* when *I_DS_* = const.) were extracted from the transfer characteristics, and the values were determined to be 0.8 V, 0.3 V, 0.5 V, and 0.7 V for HfO_2_ thicknesses of 20 nm, 40 nm, 60 nm, and 80 nm, respectively. Notably, the device with a 40 nm thick HfO_2_ layer exhibited the smallest *V_h_*, suggesting a reduced density of interface trap states at the dielectric/channel interface. These results indicate that a moderate HfO_2_ thickness of 40 nm provides more stable interfacial characteristics, contributing to improved operational reliability of the device.

The electrical performance of our IGZO TFTs was compared with previously reported devices using HfO_2_ deposited by ALD, sputtering, or solution-based methods, as well as other high-k dielectrics such as Al_2_O_3_, as shown in Table 2 [31,35,37,38,39,40]. The device with 40 nm PEALD HfO_2_ showed superior electrical performance, including a low subthreshold swing, high on/off current ratio, and stable threshold voltage. In particular, the extracted *N_t_* was lower than the reported values for IGZO TFTs using ultrathin Al_2_O_3_ (2–4 nm), which typically range from 1.7 × 10^12^ to 9.2 × 10^11^ cm^−2^·eV^−1^ [37]. These results highlight the advantages of PEALD in improving interface quality and achieving well-balanced device performance.

**Figure 7:**
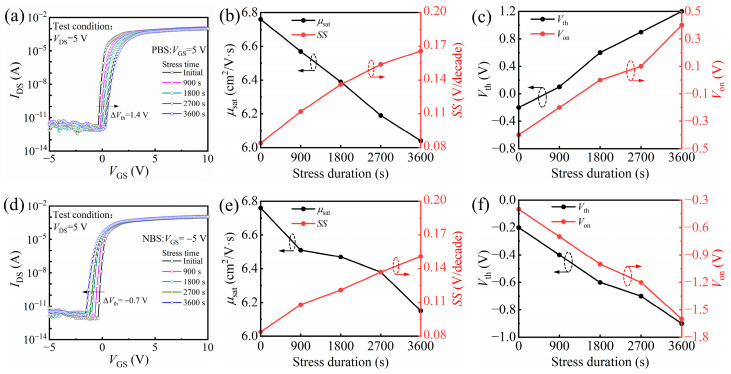
The biasing stability of IGZO-TFT based on 40 nm HfO_2_: (**a**) the transfer characteristics; (**b**) *I_on_*/*I_off_* and *SS* and (**c**) *Vth* and *V_on_* of IGZO-TFT under positive gate bias stress (PBS, *V_GS_* = +5 V); (**d**) the transfer characteristics; (**e**) *I_on_*/*I_off_* and *SS* and (**f**) *Vth* and *V_on_* of IGZO-TFT under negative bias stress (NBS, *V_GS_* = −5 V).

Both positive bias stress (PBS) and negative bias stress (NBS) tests were performed on devices with 40 nm HfO_2_ gate dielectrics, as shown in Figure 7. The gate bias was set to +5 V for PBS and −5 V for NBS, with a constant *V_DS_* of 5 V. The stress duration was 3600 s, during which the transfer characteristics were periodically measured to extract key parameters including *Vth*, *μ_sat_*, *SS*, and *V_on_*. Under PBS, the *Vth* shifted from −0.2 V to 1.2 V, and the *V_on_* shifted from −0.4 V to 0.4 V. The *μ_sat_* decreased slightly from 6.76 to 6.04 cm^2^/V·s, while the *SS* increased from 0.084 to 0.166 V/dec, indicating moderate positive charge trapping over time. Under NBS, the *Vth* shifted from −0.2 V to −0.9 V, and the *V_on_* shifted from −0.4 V to −1.6 V. Correspondingly, the *μ_sat_* decreased from 6.76 to 6.15 cm^2^/V·s, and the *SS* increased from 0.084 to 0.151 V/dec, suggesting electron detrapping or hole trapping effects. Despite the observed shifts under both stress conditions, the variations in electrical parameters remain moderate, demonstrating that the devices maintain acceptable operational stability. These results confirm the reliability of the 40 nm PEALD-grown HfO_2_ as a gate insulator for IGZO TFTs.

## 4. Conclusions

In this work, a-IGZO TFTs incorporating HfO_2_ gate insulators of varying thicknesses were systematically investigated. The self-limiting surface chemical reactions inherent to PEALD enable the precise control of thickness and uniformity, resulting in a dense surface morphology that significantly enhances the performance of TFTs. It is found that raising the HfO_2_ thickness to 40 nm leads to the highest area ratio of the (111) peak in favor of improving the dielectric constant. A *Vth* of −0.9 V, *μ_sat_* of 6.76 cm^2^/Vs, *SS* of 0.084 V/decade, and *I_on_*/*I_off_* of 1.35 × 10^9^ are obtained for IGZO TFTs with 40 nm HfO_2_. PBS and NBS tests further confirmed the stability of the 40 nm device. Although *Vth* shifts were observed over 3600 s of stress, the changes in *μ_sat_* and *SS* remained within acceptable ranges, indicating good operational reliability. It is believed that the IGZO TFT based on HfO_2_ films with suitable thickness prepared by PEALD can improve electrical performance.

## Figures and Tables

**Table 1:** Summary of the important electrical parameters of a-IGZO TFT devices with different HfO_2_ insulation thicknesses.

Thickness (nm)	*Vth* (V)	*I_on_*/*I_off_*	*μ_sat_* (cm^−2^/Vs)	*SS* (V/dec)	*N_t_* (/cm^2^)	*D_it_*	*Q_f_*
20 nm	−0.1 ± 0.15	7.05 × 10^6^	3.76 ± 0.18	0.138 ± 0.013	3.28 × 10^11^	1.26 × 10^11^	1.88 × 10^12^
40 nm	−0.2 ± 0.12	1.35 × 10^9^	6.76 ± 0.22	0.084 ± 0.010	1.14 × 10^11^	2.59 × 10^11^	2.15 × 10^12^
60 nm	−0.4 ± 0.19	1.14 × 10^9^	5.84 ± 0.21	0.114 ± 0.011	1.65 × 10^11^	2.71 × 10^11^	2.35 × 10^12^
80 nm	−1.1 ± 0.27	3.32 × 10^8^	3.25 ± 0.15	0.146 ± 0.015	1.82 × 10^11^	3.17 × 10^11^	2.76 × 10^12^

**Table 2:** Comparison of electrical performance parameters of IGZO TFTs using various gate dielectrics and deposition methods.

Dielectric Layer	*Vth* (V)	*I_on_*/*I_off_*	*μ* (cm^−2^/Vs)	*SS* (V/dec)	*N_t_* (/cm^2^)	Ref.
HfO_2_ (ALD)	0.3	8.0 × 10^6^	4.6	0.075	N.A.	[35]
HfO_2_ (ALD)	1.52	1.18 × 10^7^	16.75	0.159	1.54 × 10^12^	[31]
HfO_2_ (Sputtering)	1.1	4.3 × 10^7^	10.3	0.28	N.A.	[38]
HfO_2_ (Sputtering)	1.5	3.5 × 10^6^	30.2	0.17	N.A.	[39]
HfO_2_ (Solution)	−0.3	N.A.	85	0.140	N.A.	[40]
Al_2_O_3_ (Solution)	0.48	1.6 × 10^6^	5.40	0.068	8.5 × 10^11^	[37]
HfO_2_ (ALD)	−0.2 ± 0.12	1.35 × 10^9^	6.76 ± 0.22	0.084 ± 0.010	1.14 × 10^11^	This Work

## Data Availability

Data are contained within the article.
